# Analysis of the therapeutic effect and influencing factors on unresectable gastric cancer treated with conversion therapy

**DOI:** 10.3389/fonc.2024.1435398

**Published:** 2024-10-30

**Authors:** Saiyi Han, Shaoliang Han, Jun Qian, Mengfu Guo, Jianping Fan

**Affiliations:** ^1^ The Quzhou Affiliated Hospital of Wenzhou Medical University, Quzhou People’s Hospital, Quzhou, China; ^2^ Department of The Gastrointestinal Surgery, First Affiliated Hospital of Wenzhou Medical University, Wenzhou, China

**Keywords:** advanced unresectable gastric cancer, conversion therapy, metastasis, surgery, survival, gastrectomy, chemotherapy

## Abstract

**Background:**

Gastric cancer (GC) is one of the leading causes of cancer-related death in China, and with the extensive development of conversion therapy, the treatment of advanced unresectable gastric cancer (AUGC) patients has ushered in a new dawn. This study aimed to analyze the efficacy of conversion therapy in AUGC patients and explored the relevant factors affecting the efficacy.

**Method:**

We collected information from GC patients who received conversion therapy from this center and designed a retrospective study.

**Results:**

We collected relevant clinical data from 160 patients with AUGC. A total of 120 patients who underwent routine R0 resection were identified as conversion cases. A total of 25 patients (15.6%) achieved pCR, 92 patients (57.5%) achieved objective response rate (ORR), 140 patients (87.5%) achieved disease control rate (DCR), and 20 cases (12.5%) observed tumor progression. There were 86 patients who achieved pathological downgrading, with a total downgrading rate of 53.8%. Among the 160 patients, 37 patients (23.1%) had postoperative complications of varying degrees. A total of 72 patients (45.0%) had tumor recurrence/progression at the end of follow-up. The last chemotherapy and surgery (CST) (OR = 1.046, 95% CI 1.013–1.081, *p* = 0.006), tumor invasion (OR = 32.096, 95% CI 5.091–202.349, *p* < 0.001), and distant metastasis (OR = 7.050, 95% CI 1.888–26.323, *p* = 0.004) were independent factors influencing the efficacy of conversion therapy.

**Conclusion:**

Conversion therapy may have a good therapeutic efficacy for AUGC, and some clinical factors affect the efficacy response.

## 1 Introduction

Gastric cancer (GC) is the third most common malignancy and one of the leading causes of cancer-related deaths in China (1). Most of the patients with GC treated clinically are in the advanced stage. Surgery is the only choice to cure patients with GC, but approximately 10% of GC patients cannot undergo surgery and have to be treated with palliative care, with a median survival time of 5 to 12 months (2, 3).

In recent years, with the advancement of anti-cancer drug therapy, comprehensive treatment with drug therapy as the main approach has been adopted for stage IV GC who cannot be operated on; thereby, some of them can have the opportunity for radical surgery (i.e., R0 resection) and long-term survival after surgical resection, and this approach is also known as conversion therapy (4).

Conversion therapy was first proposed by Bismuth et al. (5), which improved the resection rate and prolonged the survival time of patients with initially unresectable liver metastases of colorectal cancer through chemotherapy and targeted therapy or immunotherapy. According to Fukuchi et al.’s research, conversion therapy may make R0 resection possible in this group of patients and significantly improve survival rates (6, 7). Some recent institutional studies have shown that the survival of stage III/IV unresectable patients undergoing conversion surgery is 37 to 56 months (8).

However, owing to the heterogeneity of tumors, not all patients can ultimately achieve R0 resection (9). Therefore, it is particularly important to accurately judge whether GC patients are suitable for conversion therapy or not and whether the best curative effect can be obtained.

This paper aimed to analyze the effectiveness of conversion therapy for unresectable GC and study the relevant clinical factors that affect treatment efficacy.

## 2 Materials and methods

### 2.1 Patient selection

We collected information from 160 GC patients who received conversion therapy at the First Affiliated Hospital of Wenzhou Medical University, China, from September 2017 to November 2023 and designed a retrospective study. All these patients underwent preoperative chemotherapy and gastrectomy. The selection strictly followed the inclusion and exclusion criteria.

The inclusion criteria were as follows: (1) all cases with histologically confirmed gastric adenocarcinoma; (2) unresectable advanced GC was determined by CT scanning and/or laparoscopic observation (AJCC/AUCC 8th staging system); (3) the presence of measurable lesions according to the Response Evaluation Criteria in Solid Tumors version 1.1 guidelines (RECIST v1.1); (4) all GC patients received drug chemotherapy with/without targeted therapy or immunotherapy; (5) the tumors were judged to have adequate response and were indicated in surgery by a multidisciplinary team (MDT), and surgical resection was performed with the patient’s informed consent; and (6) systemic examination was performed in all gastrectomy specimens.

The exclusion criteria were as follows: (1) with a history of previous systemic treatment for GC such as targeted therapy, chemotherapy, palliative gastrectomy, etc.; (2) gastric stump cancer and recurrent GC; (3) previous or co-suffering from other malignant tumors; (4) inoperable or unresected cases (i.e., laparoscopic exploration without gastrectomy and gastrojejunostomy without tumor resection); and (5) the CT images were not clear (such as motion artifacts), and because we could not use it to measure the diameter of the lesion, further efficacy determination cannot be made.

### 2.2 Conversion treatment regimens

The chemotherapy regimens included SOX (oxaliplatin plus S-1), XELOX (oxaliplatin plus capecitabine), FLOT (5-fluorouracil plus leucovorin, oxaliplatin, and docetaxel), FLOFOX (oxaliplatin plus calcium levofolinate, 5-fluorouracil), AS (paclitaxel plus S-1), TP (paclitaxel plus cisplatin), DS (S-1 plus docetaxel), and DOS (docetaxel plus S-1, oxaliplatin).

Trastuzumab was recommended for patients with HER2-positive cancers. The immunotherapy regimens included camrelizumab, pembrolizumab, sintilimab, tislelizumab, nivolumab, penpulimab, and serplulimab, and the treatment schedule and dosage were determined based on the patient’s general situation and medication application guidelines.

### 2.3 Efficacy evaluation of conversion treatment and surgery candidate selection

From every two courses after the beginning of conversion treatment, the post-treatment efficacy of chemotherapy was evaluated comprehensively by combining imaging examination, endoscopy, and laparoscopic exploration by a multidisciplinary team (MDT).

If the efficacy was evaluated as complete remission, partial remission, or stable condition, then the MDT team discussed the possibility of conversion surgery; if R0 tumor resection was possible, the so-called conversion surgery would be suggested, and the patients’ informed consent was needed.

The criteria for lymph node dissection were performed according to the Japanese Classification of Gastric Carcinoma (14th edition) guidelines (10). The 8th edition of the American Joint Committee on Cancer (AJCC) tumor–node–metastasis (TNM) classification was used for tumor staging (11). RECIST v1.1 (12) and tumor regression grade (TRG) (13) were used to assess response to conversion therapy.

### 2.4 Variables

Baseline characteristics of GC patients, including name, gender, age, hemoglobin, serum CEA, serum CA199, and distant metastasis, were collected. Chemotherapy and surgical variables, including chemotherapy regimens, immunotherapy, targeted therapy, operative procedure, R0 resection, combined resection, and the number and size of harvested lymph nodes, were collected. Tumor-related variables included preoperative staging (cTNM), postoperative pathological stage (pTNM), histological type and grading, Borrmann classification, Lauren’s classification, HER-2 status, signet ring cell carcinoma, microsatellite stability, and TRG grading.

### 2.5 Definition

The definition of a progressive unresectable tumor included the following: nodal involvement outside D1–3 stations, peritoneal cancer index (PCI) > 6 (14), bilobar or multiple hepatic metastases, and technically unresectable metastases (8).

Conversion therapy was defined as a comprehensive treatment plan (including chemotherapy, radiotherapy, targeted treatment, and immunotherapy by an MDT) aimed at patients who were unable to undergo radical resection surgery in the initial stage, transforming initially unresectable cancer into directly feasible R0 resection cancer (15). R0 resection was defined as resection of primary and metastatic tumors with no evidence of both macroscopic and microscopic malignant tumor residue at near and distant sites.

Gastrectomy included total gastrectomy and distal subtotal gastrectomy based on the tumor site, and reconstruction methods included Billroth II and Roux-en-Y. D2 and above lymphadenectomy were performed routinely for radical gastrectomy patients.

Progression-free survival (PFS) was calculated as the time from surgery to objective tumor progression, death, or the end of follow-up. Overall survival (OS) was defined as the date from the start of follow-up to tumor progression, death, or the end of follow-up.

### 2.6 Statistical analysis

Descriptive statistics were expressed as mean (SD) and median [interquartile range (IQR)]. Continuous variables were compared using the independent *t*-test or Mann–Whitney *U* test for continuous variables, while categorical variables were compared using chi-square tests or Fisher’s exact tests. The logistic model was applied to evaluate the outcome factors, and the results were expressed by odds ratio (OR) and 95% confidence interval (CI). Variables with *p* < 0.05 in the univariate analysis were included in the multivariate analysis. All analyses considered two-sided *p* < 0.05 as statistically significant. Analysis and plot tables were performed by using SPSS software version 25.0.

This study has been approved by the review committees of the institutions. All procedures were conducted in accordance with the ethical standards of the relevant committees for human experimentation (institutional and national), as well as the 1964 Helsinki Declaration and its subsequent versions (16).

## 3 Results

### 3.1 Clinicopathological characteristics

According to our inclusion and exclusion criteria, a total of 160 AGC patients ultimately were enrolled in this study. Among them, 120 patients who underwent routine R0 resection were identified as conversion cases (**Table 1**). The TNM staging of all patients before conversion therapy is shown in **Table 2**. Among them, there were 39 women (24.4%) and 121 men (75.6%), with a median age of 67.0 years (IQR 13.0 years).

**Table 1:** Conversion surgical intervention.

Variables	Overall	Groups		*t*/*z*/*χ* ^2^	*p*-value
		Good response	Poor response		
**^a^CST (days)** **Median (IQR)**	38.0 (16.0)	36.0 (12.3)	41.0 (18.0)	2.670	0.008
**Surgical procedure (*n*, %)**				0.088	0.767
Total gastrectomy	89 (55.3)	50 (54.3)	39 (56.7)		
Partial gastrectomy	71 (44.7)	42 (45.7)	29 (43.3)		
**Lymph node dissection (*n*, %)**				0.441	0.507
D2	139 (94.6)	78 (96.3)	61 (92.4)		
D3	8 (5.4)	3 (3.7)	5 (7.6)		
**Radical surgery (*n*, %)**				26.735	<0.001
R0	120 (75.0)	83 (90.2)	37 (54.4)		
Palliative	40 (25.0)	9 (9.8)	31 (45.6)		

**^a^** CST, Time interval between the last chemotherapy treatment and the surgery.

**Table 2:** The TNM factors and staging before and after conversion treatment.

Criteria	Pre-conversion treatment (*n* = 160, %)	Post-conversion treatment (*n* = 160, %)
T factor		
T0–2	0 (0)	52 (32.5)
T3	41 (25.6)	41 (25.6)
T4a	91 (56.9)	54 (33.8)
T4b	28 (17.5)	13 (8.1)
N factor		
N0	6 (3.8)	74 (46.2)
N1	7 (4.4)	27 (16.9)
N2	62 (38.8)	21 (13.1)
N3	85 (53.1)	38 (23.8)
M factor		
M0	115 (71.9)	131 (81.9)
M1	45 (28.1)	29 (18.1)
TNM stage		
0–II	0 (0)	73 (45.6)
III	106 (66.3)	49 (30.7)
IVA	9 (5.6)	9 (5.6)
IVB	45 (28.1)	29 (18.1)

All 160 cases were divided into two groups according to postoperative pathologic efficacy evaluation results (according to RECIST v1.1). Group 1 was defined as the good response group, in which patients had both CR and PR efficacy evaluation. Group 2 was defined as the poor response group, whose efficacy of patients was determined to be SD or PD (**Table 3**).

**Table 3:** The baseline clinicopathological characteristics before conversion therapy.

Variables	Overall	Groups		*t*/*z*/*χ* ^2^	*p*-value
		Good response	Poor response		
**Gender (*n*, %)**				0.282	0.596
Male	121 (75.6)	71 (77.2)	50 (73.5)		
Female	39 (24.4)	21 (22.8)	18 (26.5)		
**Age (years)** **Median (IQR)**	67.0 (13.0)	67.0 (11.7)	66.5 (15.0)	0.439	0.661
**Hemoglobin (*n*, %)**				0.027	0.869
Normal	60 (37.5)	34 (37.0)	26 (38.2)		
Abnormal	100 (62.5)	58 (63.0)	42 (61.8)		
**Serum CEA (ng/mL)** **Median (IQR)**	3.2 (6.8)	3.9 (9.1)	2.6 (4.7)	1.976	0.048
**Serum CA199 (kU/L)** **Median (IQR)**	10.2 (32.6)	10.4 (34.3)	10.1 (62.85)	0.133	0.894
**Distance metastasis (*n*, %)**				8.624	0.035
Non-metastases					
Liver	15 (9.4)	11 (12.0)	4 (5.9)		
Peritoneum	11 (6.9)	2 (2.2)	9 (13.2)		
Other sites	19 (11.9)	11 (12.0)	8 (11.8)		

For baseline characteristics (**Table 3**), the median serum CEA level was 3.9 (IQR 9.1) ng/mL in the good response group and 2.6 (IQR 4.7) ng/mL in the poor response group, with statistically different overall serum CEA levels between the two groups (*t* = 1.976, *p* = 0.048). Based on the enhanced CT images at diagnosis in all patients, we also statistically analyzed the differences in distant metastases, compared with the poor response group, and the presence of distant metastasis was lower in the good response group (26.1% vs. 30.9%), with statistical difference (*χ* ^2^ = 8.624, *p* = 0.035). There was no significant difference in sex, age, hemoglobin, and serum CA199 between the two groups (**Table 3**).

### 3.2 Chemotherapy regimens

All 160 patients underwent preoperative chemotherapy. **Table 4** shows chemotherapy- and surgery-related variables for all patients. Among them, 122 patients (77.2%) received the SOX regimen (oxaliplatin plus S-1), 9 patients (5.7%) received the FLOT regimen (5-fluorouracil, leucovorin, oxaliplatin, and docetaxel), 14 patients (8.9) received the AS regimen (paclitaxel plus S-1), and 13 patients (8.2%) had other chemotherapy regimens, including XELOX (oxaliplatin plus capecitabine), FLOFOX (oxaliplatin plus calcium levofolinate, 5-fluorouracil), TP (paclitaxel plus cisplatin), DS (S-1 plus docetaxel), and DOS (docetaxel plus S-1, oxaliplatin).

**Table 4:** Conversion treatment regimens and cycles.

Variables	Overall	Groups		*t*/*z*/*χ* ^2^	*p*-value
		Good response	Poor response		
**Chemotherapy cycle (time)** **Median (IQR)**	3.0 (1.3)	3.0 (1.0)	3.0 (2.0)	0.190	0.849
**Chemotherapy regimen (*n*, %)**				4.289	0.232
^a^SOX	122 (77.2)	70 (77.8)	52 (76.5)		
^b^FLOT	9 (5.7)	7 (7.8)	2 (2.9)		
^c^AS	14 (8.9)	5 (5.6)	9 (13.2)		
^d^Others	13 (8.2)	8 (8.9)	5 (7.4)		
**Immunotherapy (*n*, %)**				0.966	0.326
Yes	21 (13.1)	10 (10.9)	11 (16.2)		
No	139 (86.9)	82 (89.1)	57 (83.8)		
**Targeted therapy (*n*, %)**				0.005	0.942
Yes	8 (5.0)	4 (4.3)	4 (5.9)		
No	152 (95.0)	88 (95.7)	64 (94.1)		

a SOX: oxaliplatin plus S-1.

b FLOT: 5-fluorouracil plus leucovorin, oxaliplatin, and docetaxel.

c AS: paclitaxel plus S-1.

d Others: including other chemotherapy regimens beyond the above: ① XELOX (oxaliplatin plus capecitabine); ② FLOFOX (oxaliplatin plus calcium levofolinate, 5-fluorouracil); ③ TP (paclitaxel plus cisplatin); ④ DS (S-1 plus docetaxel); ⑤ DOS (docetaxel plus S-1, oxaliplatin). Clinicians determined the appropriate dose and treatment schedule.

Univariate analysis showed that there was no significant statistical difference between the two groups in different chemotherapy regimens (*χ* ^2^ = 4.289, *p* = 0.232). A total of 21 (13.1%) patients received combined immunotherapy. The proportion of patients receiving targeted therapy between the two groups was not significantly different (4.3% vs. 5.9%). The median cycle of chemotherapy was 3.0 (IQR 1.3) times.

### 3.3 Surgical procedures and CST

In **Table 1**, according to the different locations of the tumors, 89 patients underwent total gastrectomy (55.3%) and 71 patients underwent distal gastrectomy (44.7%). The routine lymphadenectomy was D2 or above, and D2 resection accounted for 94.6% of all cases. A total of 23 patients required combined resection procedures, including hepatectomy in 5 patients, splenectomy in 11 patients, cholecystectomy in 2 patients, and intestinal resection in 2 patients. Of all 120 conversion cases, 83 (90.2%) were in the good response group and 37 (54.4%) were in the poor response group.

The median time interval between the last chemotherapy and surgery (CST) in the good response group was 36.0 (IQR 12.3) days, while in the poor response group, it was 41.0 (IQR 18.0) days. There was a statistically significant difference between the two groups (*z* = 2.670, *p* = 0.008) (**Table 1**).

We observed the postoperative complications of all patients, and the results are shown in **Table 5**: Among the 160 patients, 37 patients (23.1%) had postoperative complications of varying degrees. The most common complications were postoperative bleeding in 15 cases (40.5%) and pancreatic fistula in 7 cases (18.9%). Among them, there were two cases (5.4%) of postoperative abdominal bleeding combined with intestinal obstruction and one case (2.7%) of pancreatic fistula combined with abdominal abscess. In addition, five patients had Clavien-Dindo grade ≥III (17), including three patients with postoperative hemorrhage who underwent unplanned secondary operation (or an endoscopic procedure), one patient with pyopneumopneumothorax entering the ICU for treatment due to dyspnea, and one patient with postoperative hemorrhage complicated with intestinal obstruction entering the ICU after secondary hemostasis operation. Moreover, all the remaining patients with Clavien-Dindo grade <III improved after conservative treatment. All 37 patients were finally discharged smoothly after conservative treatment.

**Table 5:** Postoperative complications after conversion surgery.

Postoperative complications	Cases (*n* = 160)	Percentage (%)
Abdominal hemorrhage	15	40.0
Anastomotic fistula	2	5.4
Anastomotic stenosis	1	2.7
Bowel obstruction	4	10.8
Incision infection	2	5.4
Intra-abdominal abscess	1	2.7
Lymphatic fistula	3	8.1
Pancreatic fistula	7	18.9
Bile leakage	2	5.4
Pyopneumothorax	1	2.7
Bacteremia	2	5.4

### 3.4 Pathological results and response to therapeutic therapy

As shown in **Table 6**, univariate analysis showed that there was significant difference between the two groups in vascular involvement (*χ* ^2^ = 10.676, *p* < 0.001), neurological involvement (*χ* ^2^ = 29.975, *p* < 0.001), tumor invasion (ypT) (*χ* ^2^ = 42.531, *p* < 0.001), lymph node metastasis (ypN) (*χ* ^2^ = 13.114, *p* < 0.001), specific number of lymph node metastases (*z* = 3.668, *p* < 0.001), and distant metastasis (ypM) (*χ* ^2^ = 12.970, *p* < 0.001). Compared with the diagnosis results, the proportion of patients with postoperative pathological confirmation of distant metastasis was significantly reduced in the total population [45 cases (28.1%) vs. 29 cases (18.1%)] (**Tables 3**, **7**). The proportion of distant metastasis in the good response group was significantly lower than that in the poor response group [8 (8.7%) vs. 21 (30.9%)].

**Table 6:** Clinicopathological characteristics of unresectable gastric cancer.

Variables	Overall	Groups		*t*/*z*/*χ* ^2^	*p*-value
		Good response	Poor response		
**Number of lymph nodes** **Median (IQR)**	1.0 (6.8)	0 (2.0)	3.0 (10.0)	3.668	<0.001
**Gross type (*n*, %)**				0.002	>0.999
Ulcerative type	111 (82.2)	55 (82.1)	56 (82.4)		
Non-ulcer type	24 (17.8)	12 (17.9)	12 (17.6)		
**Signet-ring cell carcinoma (*n*, %)**				2.873	0.090
Yes	22 (13.8)	9 (9.8)	13 (19.1)		
Non	138 (86.3)	83 (90.2)	55 (80.9)		
**^a^Lauren class (*n*, %)**				1.416	0.493
Diffuse type	51 (45.5)	25 (47.2)	26 (44.1)		
Intestinal type	30 (26.8)	16 (30.2)	14 (23.7)		
Mixed type	31 (27.7)	12 (22.6)	19 (32.2)		
**Histopathology (*n*, %)**				2.571	0.277
Poorly differentiated	95 (72.0)	45 (70.3)	50 (73.5)		
Moderately	32 (24.2)	18 (28.1)	14 (20.6)		
Well	5 (3.8)	1 (1.6)	4 (5.9)		
**^b^Vascular invol (*n*, %)**				10.676	0.001
Positive	61 (38.4)	25 (27.5)	36 (52.9)		
Negative	98 (61.6)	66 (72.5)	32 (47.1)		
**^c^Neurological invol (*n*, %)**				29.975	<0.001
Positive	77 (48.4)	27 (29.7)	50 (73.5)		
Negative	82 (51.6)	64 (70.3)	18 (26.5)		
**HER-2 status (*n*, %)**				0.023	0.879
Positive	49 (34.5)	27 (35.1)	22 (33.8)		
Negative	93 (65.5)	50 (64.9)	43 (66.2)		
**^d^MSI status (*n*, %)**				0.054	>0.999
pMMR	124 (95.4)	68 (95.8)	56 (94.9)		
dMMR	6 (4.6)	3 (4.2)	3 (5.1)		
**^e^EGFR (*n*, %)**				1.148	0.284
Negative	10 (14.1)	7 (20.0)	3 (8.3)		
Positive	61 (85.9)	28 (80.0)	33 (91.7)		
**ypT (*n*, %)**				42.531	<0.001
ypT0–2	52 (32.5)	49 (53.3)	3 (4.4)		
ypT3–4	108 (67.5)	43 (46.7)	65 (95.6)		
**ypN (*n*, %)**				13.114	<0.001
ypN0–1	101 (63.1)	69 (75.0)	32 (47.1)		
ypN2–3	59 (36.9)	23 (25.0)	36 (52.9)		
**ypM (*n*, %)**				12.970	<0.001
ypM0	131 (81.9)	84 (91.3)	47 (69.1)		
ypM1	29 (18.1)	8 (8.7)	21 (30.9)		

^a^Lauren classification (Lauren class); ^b^Vascular involvement (Vascular invol); ^c^neurological involvement (Neurological invol); ^d^microsatellite instability (MSI); ^e^Epidermal growth factor receptor (EGFR).

**Table 7:** Postoperative pathological confirmed distant metastasis.

Distant metastases	Cases (*n* = 29)	Percentage (%)
Liver	6	20.7
No.16 lymph node	3	10.3
Peritoneum	10	34.5
Other abdominal organs	10	34.5

The tumor pathological response in all cases was evaluated according to the evaluation criteria for solid tumor efficacy (RECIST v1.1) and TRG grading (as shown in **Table 8**). A total of 160 enrolled patients were classified into four categories based on the RECIST v1.1 efficacy evaluation level: complete response (pCR), partial response (pPR), stable disease (pSD), and progression disease (pPD). Among them, 25 patients (15.6%) achieved pCR, 92 patients (57.5%) achieved objective response rate (ORR), 140 patients (87.5%) achieved disease control rate (DCR), and 20 cases (12.5%) observed tumor progression. Among them, in 37 patients, although the total diameter of their target lesion was less than 30%, and the efficacy of RECIST v1.1 was assessed as SD, their postoperative pathology suggested radical (R0) resection. According to the TRG, the patients were also classified into four categories (0–3 levels), and no or fewer tumor residues were found in postoperative pathology (0–1 levels) in 60 cases (37.5%).

### 3.5 Down-staging and survival outcomes

Changes in TNM staging between before and after conversion therapy for all patients are listed in **Table 2**. All patients were enrolled with a T-stage of T3–4, while 52 patients (32.5%) dropped below T3 after treatment. The proportion of N2–3 patients decreased from 91.9% (*n* = 147) before treatment to 36.9% (*n* = 59). Sixteen patients (10.0%) experienced a decrease from M1 to M0. All patients were in stages III and IV before conversion treatment, but after treatment, 45.6% (*n* = 73) were in stages 0–II, of which 57 cases (62.0%) were in the good response group, and all cases that dropped to stage 0 (*n* = 23, 25.0%) were in this group. Comparing the TNM staging (stages 0–IV) of all patients before and after treatment, a total of 86 patients achieved pathological downgradation, with a total downgradation rate of 53.8%.

To further explore the effect of conversion therapy on patient survival outcomes, all patients were followed up after surgery. Based on whether R0 resection was performed, all 160 patients were divided into two groups for subgroup analysis; that is, patients who underwent R0 resection were defined as the conversion group, while patients who did not undergo R0 surgery were defined as the non-conversion group. The median follow-up time for all patients was 14.7 (IQR 14.6) months, and the median PFS and OS were 22.2 and 26.0 months in the conversion group, respectively, whereas the median PFS and OS in the non-conversion group were 7.7 and 13.2 months, respectively. At the end of follow-up, a total of 72 patients (45.0%) experienced tumor recurrence/progression, and the proportion of recurrence/progression in the conversion group (39.2%) was significantly lower than that (62.5%) in the non-conversion group. Among patients who achieved pCR, the postoperative recurrence was observed in five cases (**Table 8**).

**Table 8:** Response results and survival outcomes of conversion therapy efficacy.

Assessment	Cases (*n* = 160)	Percentage (%)
Postoperative pathological efficacy		
^a^pCR	25	15.6
^b^pPR	67	41.9
^c^pSD	48	30.0
^d^pPD	20	12.5
^e^TRG grade		
0	25	15.6
1	35	21.9
2	60	37.5
3	40	25.0
Recurrence		
Yes	72	45.0
No	88	55.0

^a^Pathological complete remission (PCR); ^b^Pathological partial remission (pPR); ^c^Pathological stable condition pSD; ^d^Pathological progressive disease pPD; ^e^Tumor Regression Grade (TRG).

### 3.6 Multivariate logistic regression analysis of conversion therapy

Univariate analysis showed significant differences in serum CEA, the interval between the last chemotherapy and surgery (CST), number of lymph node metastases, radical surgery, neural involvement, vascular involvement, tumor invasion, lymph node metastasis, and distant metastasis, but no significant differences were observed in gender, age, serum CA199, chemotherapy regimen, gastric surgery, lymph node dissection, HER-2 status, and signet ring cell carcinoma.

To further explore the independent influencing factors for conversion therapy efficacy, we included meaningful indicators from univariate analysis as covariates in multivariate logistic regression analysis. The results showed that the following factors had a statistically significant impact on the response to conversion therapy efficacy: (1) the longer interval between the last chemotherapy and surgery (CST), the higher the probability of low or no response (OR = 1.046, 95% CI 1.013–1.081, *p* = 0.006); (2) the tumor invasion of ypT3-4 increasing the risk of low or no response to conversion therapy (OR = 32.096, 95% CI 5.091–202.349, *p* < 0.001); and (3) distant metastasis (M1) affecting the patients’ response to conversion therapy (OR = 7.050, 95% CI 1.888–26.323, *p* = 0.004) (**Table 9**).

**Table 9:** Multivariate logistic regression analysis on conversion surgery.

Variables	Control group	S.E.	Wald	*p*-value	OR	95% CI	
						Lower limit	Upper limit
**Serum CEA**	−0.018	0.011	2.592	0.107	0.982	0.961	1.004
**CST**	0.046	0.017	7.468	0.006	1.047	1.013	1.082
**Number of lymph node**	0.076	0.057	1.789	0.181	1.079	0.965	1.206
Radical surgery							
R0*	0.160	0.833	0.037	0.848	1.173	0.229	6.010
Palliative							
Nerve involvement							
Negative*	0.773	0.492	2.470	0.116	2.165	0.826	5.676
Positive							
Vascular involvement							
Negative*	−0.082	0.570	0.020	0.886	0.922	0.302	2.816
Positive							
ypT							
0–2*	3.490	0.950	13.495	<0.001	32.789	5.094	211.153
3–4							
ypN							
0–1*	−0.278	0.759	0.134	0.714	0.757	0.171	3.356
2–3							
ypM							
0*	1.848	0.860	4.618	0.032	6.346	1.176	34.237
1							

Control (Reference) group*.

## 4 Discussion

The argument for conversion therapy of GC was first proposed by Nakajima et al. (18) more than 20 years ago, who suggested that R0 resection could be performed after chemotherapy for unresectable GC and that such patients had a longer OS. In this study, we reviewed the clinical and pathological characteristics of 160 patients before and after receiving conversion therapy and evaluated the efficacy response of all patients according to the RECIST V.1.1 standard. Our research indicates that some factors have a significant impact on the effectiveness of conversion therapy, and the better the response to conversion therapy, the benefit of conversion therapy on patients’ postoperative survival outcomes becomes significant.

In this study, 120 patients (75.0%) were eligible for radical resection after conversion therapy. We also compared the TNM staging of all patients before and after treatment, and the total down-staging rate reached 53.8%. At present, there are many international reports on the effectiveness of conversion therapy. According to the report, the proportion of patients who can undergo surgery after conversion is 25%–80%, and the R0 resection rate of surgical patients is between 15% and 65% (15, 19, 20). Comparing these reports indicated that our study had a high conversion success rate, and more than half of them could achieve pathological regression or even complete pathological regression after conversion treatment.

An international study published in *The Lancet* in 2021 (21) showed that among 1,337 patients undergoing GC surgery from 263 hospitals, there were 612 (46.3%) postoperative complications. Among them, major complications (Clavien-Dindo grade ≥III) occurred in 192 cases (14.5%). This study provided a unique prospective dataset for the occurrence of postoperative complications and outcomes in patients undergoing GC surgery worldwide. In our study, the incidence of early complications after conversion surgery was 23.1%, with five cases (3.1%) having a Clavien-Dindo grade ≥III, and the remaining complications were required only relevant conservative treatment to improve. This result indicated that our conversion surgery could ensure a certain level of safety and feasibility, and the early postoperative quality of life for cancer patients would not bring additional burdens.

A clinical study by Xue et al. (22) showed that the R0 removal rate after conversion in patients with stage IV GC was 59.6%, and the postoperative ORR could reach 61.7%. Their results proved that conversion therapy could provide a higher possibility of stage I cure for cancer patients. However, their study did not show a significant relationship between conversion surgery and long-term outcomes in patients with AGC. In this study, the overall objective response rate (ORR) of the population was 57.5%, and the final pCR rate was 15.6%. Follow-up analysis of the prognosis of all cases showed that the median PFS and median OS of 120 cases receiving conversion therapy were significantly higher than those of non-conversion therapy, which proved that conversion therapy had a significant benefit on the survival outcome and long-term prognosis of AGC patients. The better the pathological efficacy grade after conversion surgery, the higher the survival benefit of patients.

Admittedly, there were also some cases in our study that did not benefit from conversion therapy. These cases did not receive good pathological benefits after conversion therapy, and postoperative pathological examination often reveals tumor progression, which eventually leads to poor survival rate. This indicates that there are individual differences in conversion therapy, and not all patients can benefit from it. Different physiological, pathological, or clinical factors may affect the performance of therapeutic efficacy, thereby affecting the prognosis of patients.

Understanding the depth of primary tumor invasion and lymph node involvement is of great significance for the diagnosis and treatment of GC, and its impact on the efficacy and prognosis of conversion therapy cannot be ignored. A retrospective study by Sagawa et al. (23) showed that the median OS of stage IV GC patients undergoing gastrectomy was 40.0 months, and pathological tumor depth and lymph node involvement were important prognostic factors for GC by statistical analysis. Other studies have also shown that ypT staging and lymph node involvement have a significant effect on the efficacy of conversion therapy (24, 25). In this study, we observed that the ypT staging was an independent risk factor for conversion therapy response in GC patients, and patients with postoperative pathological staging ypT3-4 had relatively poor efficacy, but the correlation between lymph node metastasis and efficacy response was not yet clear, and further research is needed to confirm.

One of the reasons why certain cancers cannot be resected is the distant metastasis of the tumor. Among the 160 patients in this study, a total of 29 patients (18.1%) had distant metastases confirmed by pathology after surgery. We analyzed the impact of distant metastasis on the response to conversion therapy, and the results showed that distant metastasis was an independent risk factor affecting the response to conversion therapy. A study (26) based on the National Cancer Database (NCDB) in the United States showed that 26% of GC patients had liver metastases, 43% had peritoneal metastases, and 20% had distant lymph node metastases. This is similar to our findings; the most common sites of metastasis in the 29 patients with distant metastases were peritoneum (34.5%) and liver (20.7%). A randomized GERCOR study showed that the effective rate of oxaliplatin and FU/LV infusion (FOLFOX) regimen for liver metastasis of colorectal cancer was 54%, which demonstrated the effectiveness of preoperative chemotherapy for liver metastasis regression (27). CheckMate 649 study showed that the median OS of GC patients combined with peritoneal metastasis after immunochemotherapy was worse than that of patients without peritoneal metastasis (9.5 months vs. 15.6 months), which confirmed that peritoneal metastasis has a significant impact on the prognosis of GC patients (28). The prognosis of patients is often influenced by a combination of multiple factors, and the above studies have not demonstrated a direct association between distant metastasis and the response to conversion therapy for GC.

Chen et al. (29) emphasized adaptive immune nonresponse in gastric signet ring cell carcinoma (GSRCC) and the mediating role of CXCL13 in the tumor immune microenvironment (TIME). In the study of Liang et al. (30), there was a significant correlation between the presence of signet-ring cell carcinoma and tumor recurrence. However, histological signet-ring cell carcinoma did not prove to have an effect on the conversion effect in this study (*χ* ^2^ = 2.873, *p* = 0.090), and further studies on the heterogeneity and microenvironment features of signet-ring cell carcinoma are needed to obtain better conversion effect.

In our study, there were still some patients who did not benefit from the conversion therapy, which may be related to the specific biological behavior and sensitivity of GC to conversion drugs. Some GC-related studies have conducted more in-depth studies on the efficacy and mechanism of drugs using RECIST v.1.1 as the efficacy standard and explored the impact of different systems on ORR (31–33). The German phase II AIOFLOT3 trial investigated the efficacy of conversion therapy with the FLOT regimen in 238 stage IV GC patients from 52 centers, and the results showed that patients who underwent conversion surgery had better survival outcomes than those who did not undergo conversion surgery (34). In addition, relevant studies have shown that the combination of immune checkpoint inhibitors (ICIs) and chemotherapy can exert synergistic anti-tumor activity by modulating the immune system or reshaping the tumor microenvironment (15). The study of Janjigian et al. (35) showed that nivolumab was the first PD-1 inhibitor to show superior OS, PFS benefits, and an acceptable safety profile in patients with previously untreated advanced GC. Since then, chemotherapy combined with the nivolumab regimen has become the first-line treatment for unresectable GC. In a prospective study by Li et al. (36), the combination of camrelizumab, apatinib, and S-1 ± oxaliplatin was used to treat cT4a/bN+ GC, and it promoted active immune cell infiltration by reducing tumor mutation/neoantigen burden (TMB/TNB) to remodel the immune microenvironment and induce an anti-tumor immune response.

The result showed that the MPR+(MPR&CPR) rate was 27.8%, and 13 (76.5%) of 17 patients with R0 resection had no recurrence. This indicates that this new adjuvant/conversion therapy regimen is safe and effective. From the above results, it can be concluded that different conversion therapy regimens also have different therapeutic effects on patients with advanced GC. Our study did not show statistically significant differences in the use of different chemotherapy regimens and immune and targeted therapies. Further clinical research studies are needed to determine how to choose the best conversion plan, how to improve the effectiveness of conversion therapy, and how to obtain better survival benefits.

The choice of timing for conversion surgery has a significant impact on the response and prognosis of conversion therapy. However, some GC patients may miss the optimal surgical opportunity due to the toxic side effects of chemotherapy drugs, tumor-related complications, and the patient’s own nutritional status, which is obviously not conducive to the prognosis of patients. A study by Sagawa et al. (37) demonstrated that delayed conversion surgery for stage IV cancer could improve the 3-year survival rate. Some GC conversion studies suggested an interval of 4 to 6 weeks as the surgical interval, but there is no clear evidence to support the correctness of this interval (38, 39). Ling et al.’s study (40) showed that patients with a surgical interval >6 weeks had poorer OS compared to patients at 4–6 weeks, but there was no significant difference in disease-free survival between the two groups of patients. Overall, there is no consensus on the appropriate surgical interval (CTS) for patients with AGC after completion of chemotherapy. In this study, the median CST of patients in the responding group was 36.0 (31.0, 43.3) days, while in the non-responding group, it was 41.0 (34.0, 52.0) days. Multivariate analysis showed that CST was a risk factor for relatively poor response to conversion therapy, and the longer the CST, the higher the risk of poor response. More research is needed to confirm this result.

The main methods for evaluating the timing of chemotherapy surgery for conversion cases are imaging examination methods and laparoscopic exploration. However, in our research analysis, we found significant differences between preoperative tumor radiological staging and postoperative tumor pathological staging in some patients. Some suspected tumor sites in preoperative radiological abnormalities were not found to have evidence of tumor presence in postoperative pathological examination.

Some related studies compared the radiological consistency between baseline CT scanning and histopathological staging, and the results showed that the T-stage was 69%–88%, and the N-stage was 51%–71% (30). Cascone et al. (41) proposed the term “Lymph Node Immune Flare (NIF)” in their study, suggesting that this radiological pathological difference in lymph nodes may be due to inflammation after chemotherapy and immunotherapy, rather than true tumor residue. This warns us that the imaging staging of tumors is not entirely accurate, and it is necessary to combine the patients’ clinical symptoms with various technical means to accurately select the best surgical timing and achieve the best conversion effect.

The main limitation of this study is that it is a single-center retrospective study with a small sample size, and owing to the heterogeneity of tumors and significant inconsistencies in chemotherapy regimens and surgical resection ranges, it is difficult to explore the impact of some variables on the efficacy of conversion therapy, and some arguments still need further research to verify.

## 5 Conclusion

Our study suggests that effective conversion therapy can improve the R0 resection rate of advanced GC and provide better survival outcomes for these patients. High T-stage primary tumor with infiltration, distant metastasis, and inappropriate timing of conversion surgery may have a significant adverse effect on the therapeutic response of GC patients to conversion therapy. This study is expected to provide reference for subsequent research on conversion therapy for GC.

## Data Availability

The raw data supporting the conclusions of this article will be made available by the authors, without undue reservation.
